# Role of c-Fos in orthodontic tooth movement: an in vivo study using transgenic mice

**DOI:** 10.1007/s00784-020-03503-1

**Published:** 2020-08-15

**Authors:** Maximilian G. Decker, Cita Nottmeier, Julia Luther, Anke Baranowsky, Bärbel Kahl-Nieke, Michael Amling, Thorsten Schinke, Jean-Pierre David, Till Koehne

**Affiliations:** 1 Department of Orthodontics, University Medical Center Hamburg-Eppendorf, Martinistr. 52, 20246 Hamburg, Germany; 2 Institute of Osteology and Biomechanics, University Medical Center Hamburg-Eppendorf, Hamburg, Germany

**Keywords:** Orthodontic tooth movement, c-Fos, Mechanical stimulation, Bone remodelling, Root resorption

## Abstract

**Objectives:**

The transcription factor *c-Fos* controls the differentiation of osteoclasts and is expressed in periodontal ligament cells after mechanical stimulation in vitro. However, it is unclear how *c-Fos* regulates orthodontic tooth movement (OTM) in vivo. The aim of this study was therefore to analyse OTM in transgenic mice with overexpression of *c-Fos*.

**Materials and methods:**

We employed *c-Fos* transgenic mice (*c-Fos* tg) and wild-type littermates (WT) in a model of OTM induced by Nitinol tension springs that were bonded between the left first maxillary molars and the upper incisors. The unstimulated contralateral side served as an internal control. Mice were analysed by contact radiography, micro-computed tomography, decalcified histology and histochemistry.

**Results:**

Our analysis of the unstimulated side revealed that alveolar bone and root morphology were similar between c*-Fos* tg and control mice. However, we observed more osteoclasts in the alveolar bone of *c-Fos* tg mice as tartrate-resistant acid phosphatase (TRAP)-positive cells were increased by 40%. After 12 days of OTM, *c-Fos* tg mice exhibited 62% increased tooth movement as compared with WT mice. Despite the faster tooth movement, *c-Fos* tg and WT mice displayed the same amount of root resorption. Importantly, we did not observe orthodontically induced tissue necrosis (i.e. hyalinization) in *c-Fos* tg mice, while this was a common finding in WT mice.

**Conclusion:**

Overexpression of c-Fos accelerates tooth movement without causing more root resorption.

**Clinical relevance:**

Accelerated tooth movement must not result in more root resorption as higher tissue turnover may decrease the amount of mechanically induced tissue necrosis.

**Electronic supplementary material:**

The online version of this article (10.1007/s00784-020-03503-1) contains supplementary material, which is available to authorized users.

## Introduction

Orthodontic tooth movement (OTM) is a prime example of mechanically induced bone remodelling. The transduction of mechanical stimuli into differentiation and activity of bone-building osteoblasts and bone-degrading osteoclasts is regulated by a variety of genetic and epigenetic factors [1]. This explains why the biological response to mechanical forces can significantly vary from one patient to another [2]. In fact, significant differences have been observed among patients with regard to the velocity of OTM [3, 4] or the occurrence of adverse effects such as root resorption [5]. Since these differences require a patient-specific management in orthodontic care, there is a clear need towards a deeper understanding of the biological principles of tooth movement.

Recently, genetically modified mice were used to study the genetic basis of tooth movement [6–11]. Whereas larger animal models are easier to handle, only mice offer the possibility to analyse the role of single genes during OTM in vivo. Mouse models are therefore a valuable approach to decrease the gap that exists between our knowledge from in vitro studies and the small list of genes that are actually known to regulate OTM in vivo.

Of particular interest in this regard is *c-Fos*, a member of the AP-1 transcription factor family. *C-fos* is activated in osteoclast precursors and is required for osteoclast differentiation [12]. Deletion of *c-Fos* in mice leads to osteopetrosis, a phenotype characterized by abnormally high bone mass due to disturbed bone resorption [13, 14]. Conversely, overexpression of *c-Fos* in mice leads to the development of chondrogenic tumours [15]. These tumours are also evident in the occipital bones of the skull, which ossify through endochondral ossification. However, it is unclear whether *c-Fos* overexpression also affects the jaw bones, which ossify through intramembranous ossification.

Interestingly, *c-Fos* was also identified as a key mechanosensor in early gene transcription after mechanical loading [16]. In fact, numerous in vitro studies have demonstrated that mechanical forces result in an upregulation of *c-Fos* in various cell types including osteocytes [17, 18], osteoblasts [19, 20], and periodontal cells [21–23]. In particular, compression or extension of periodontal ligament cells leads to an induction of *C-FOS* on the RNA and protein level [21–23]. Although these studies clearly suggest that *c-Fos* plays a key role in OTM, it remains to be established whether and how *c-Fos* controls OTM in vivo.

The aim of this study was therefore to analyse the role of *c-Fos* in OTM by using mice with overexpression of *c-Fos* (*c-Fos* tg) and control littermates in a mouse model of OTM.

## Materials and methods

### Mice

**Fig. 1:**
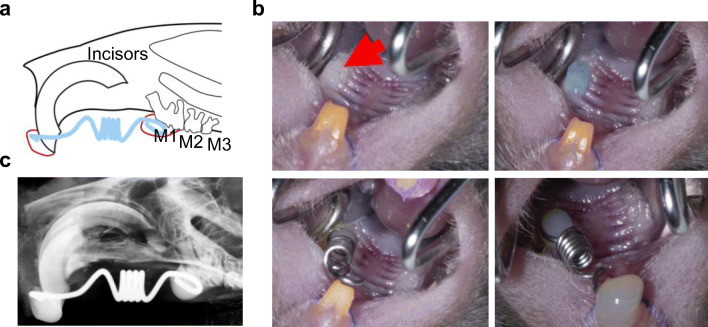
Overexpression of *c-Fos* accelerates orthodontic tooth movement in mice. **a** Schematic drawing of the OTM model. The Nitinol spring (blue) was bonded to the incisors and to the left first molar (M1) with a light-curing composite (red circles). **b** Photographs of the bonding procedure. The first molar (red arrow) was dried (upper left panel) and etched (upper right panel) before the mesial end of the spring was bonded (lower left panel). After activation of the spring with a force of 35 centinewton, the distal end of the spring was bonded to the incisors (lower right panel). **c** Contact radiography showing the activated nitinol spring

*C-fos* transgenic mice (*c-Fos* tg) were maintained on a C57BL/6J background and fed a soft rodent diet. The transgenic mice overexpress the *c-Fos* gene under the control of the glucocorticoid- and heavy metal-inducible human metallothionein promoter, which is ubiquitously expressed [24–27]. Wild-type littermates (WT) served as controls and only females were used. The orthodontic appliance was applied to the mice while they were under anaesthesia and at 10 weeks of age (Fig. 1a). After 12 days of OTM, all mice were euthanized by CO_2_ inhalation. Animal treatment procedures were approved by the commission for animal welfare (Behörde für Gesundheit und Verbraucherschutz der Hansestadt Hamburg, Nr. 121/16).

### Orthodontic appliance

All mice were anaesthetized by intraperitoneal injection with 10 ml/kg anaesthetic mixture (40 mg/kg bw ketamine-S, xylazine 2% 16 mg/kg bw, heparin 40,000 IE/kg bw in 0.9% NaCl). The mice were carefully fixed on a modified miniature lathe (Unimat 3, Emco, Wiener Neudorf, Austria). The cheeks were gently spread with a specially designed carriage holder and the upper jaw was carefully fixed to the operating table using a loop made of suture material (Vicryl, Ethicon Inc, New Jersey, USA). The incisors of the mandible were then inserted into the eyelet of an orthodontic rubber chain (Elasto-Force, Dentaurum, Ispringen, Germany) and fixed without tension. The tooth surfaces of the first left molar and both maxillary incisors were etched with 37% phosphoric acid gel (HS-etchgel 37%, Henry Schein Dental, Langen, Germany) (Fig. 1a and b). After 30 s, the gel was removed with microbrushes and the tooth surfaces were cleaned and dried successively with ethanol and specially prepared paper tips. Bonding (Scotchbond, 3 m Espe, Neuss, Germany) was applied and polymerized (Fig. 1b). The spring was placed with its distal end on the first molar and fixed with a light-curing composite (Estelite Flow Quick, Tokuyama Dental Corp., Tokyo, Japan). To activate the spring, the operating table was moved parallel to the planned force direction until the desired force of 35 centinewton was reached (tension gauge, Dentaurum, Ispringen, Germany) [9]. The mesial part of the spring was then fixed to both maxillary incisors with a light-curing composite (Estelite Flow Quick, Tokuyama Dental Corp., Tokyo, Japan) (Fig. 1b and c). After surgery, mice were transferred to a heat mat and monitored until they fully recovered from anaesthesia.

### Micro-CT and histological analysis

After 12 days of OTM, all mice were euthanized and fixed in 4% PB-buffered formaldehyde for 24 h. The skulls were removed and analysed by contact radiography using a Faxitron X-ray cabinet (Faxitron X-ray Corp., Wheeling, IL, USA). X-ray microtomography (μCT) of the skulls was performed with a μCT 40 scanner (Scanco Medical, Bassersdorf, Switzerland). Images were constructed at a spatial resolution of 15 μm. Exposed surfaces of the roots were highlighted on μCT images using Photoshop (Photoshop Cs 6, Adobe Systems Inc., USA). Orthodontic tooth movement and alveolar bone loss were evaluated on μ-CT images using ImageJ 1.52 (National Institutes of Health, Bethesda, MD, USA). Orthodontic tooth movement was defined as the shortest distance between the crowns of the maxillary first and second molars measured on μ-CT cross-sections. Alveolar bone loss was defined as the area of the exposed root surface measured on μ-CT three-dimensional reconstructions. For histology, skulls were decalcified for 14 days in Usedecalc (MEDITE Medical GmbH, Burgdorf, Germany), dehydrated in ascending alcohol concentrations, and embedded in paraffin. Four-micrometre-thick sections were cut on a microtome (Supercut 2050, Reichert-Jung, Leica Microsystems GmbH, Wetzlar, Germany). Slides were deparaffinized in xylene and stained with toluidine blue (1%, pH 4.5) for 30 min. For TRAP staining, slides were deparaffinized and stained with TRAP for 120 min at 37 °C (50 ml TRAP solution: 5 mg Naphtol-AS-MX phosphate dissolved in 500 μl dimethyl formamide, 30 mg Fast Red Violet; 40 mM sodium acetate and 10 mM sodium tartrate as buffer). Histomorphometric quantification was performed using the Osteo-Measure histomorphometry system (Osteometrics, Atlanta, GA, USA).

### Statistics

The statistical analysis of the data as well as their graphic representation was carried out with the software GraphPad PRISM (GraphPad Software, San Diego, USA). A two-sided *t* test was used for statistical testing of independent samples. ANOVA with Bonferroni post hoc test was used for multi-group comparisons. *P* values below 0.05 were considered statistically significant. All graphs show mean values with standard deviations.

## Results

**Fig. 2:**
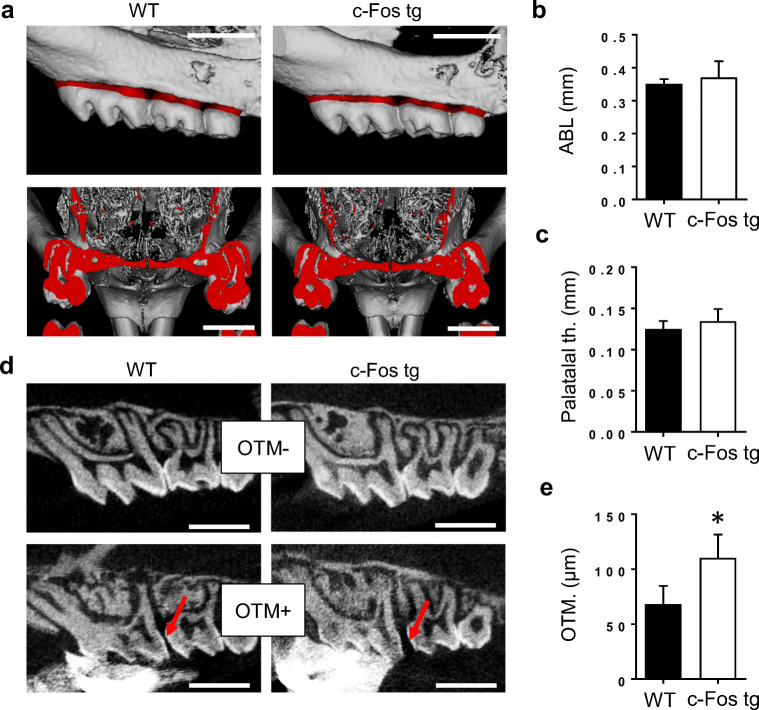
Micro-CT analysis of alveolar bone, tooth structure and OTM. **a** Micro-CT scanning of maxillary molars from 12-week-old wild-type (WT) and *c-Fos* transgenic (c-Fos tg) mice. Alveolar bone loss (highlighted in red) was measured on 3D reconstructions of teeth that were not subjected to OTM (upper panels). Palatal thickness was measured on cross-sections of the palate (lower panels). Scale bars = 3 mm. **b**, **c** Quantification of the alveolar bone loss (**b**) and palatal thickness (**c**) of 12-week-old WT and *c-Fos* tg mice. *n* ≥ 3. *P < 0.05, versus WT. **d** Cross-sections based on micro-CT scans of untreated (OTM−) and treated (OTM+) maxillary molars of wild-type (WT) and *c-Fos* transgenic mice (*c-Fos* tg). The mechanical loading created a gap between the first and second molar (red arrows). Scale bars = 1 mm. **e** Quantification of the smallest distance between the first and second molar as a surrogate measurement for OTM in 12-week-old wild-type (WT) and *c-Fos* tg mice. *n* = 4. *P < 0.05, versus wild-type

We first examined the teeth and alveolar bone of *c-Fos* tg and WT mice without orthodontic tooth movement (OTM−) using μ-CT imaging (Fig. 2). We observed that tooth morphology, alveolar bone, and palatal bone of *c-Fos* tg were similar to those of controls (Fig. 2a). In fact, quantification of alveolar bone loss (ABL) and palatal thickness revealed no differences between *c-Fos* tg and WT mice (Fig. 2b and c). We next determined the effect of orthodontic tooth movement (OTM+) in *c-Fos* and control mice (Fig. 2d, lower panels). We observed that OTM resulted in a separation of the 1st and 2nd molar crowns, which was significantly wider in *c-Fos* tg mice as compared with those of controls. In fact, quantification of the shortest distance between the 1st and 2nd molars revealed 62% increased tooth movement in *c-Fos* tg mice (Fig. 2e). Taken together, these first analyses suggest that *c-Fos* overexpression increases OTM and that this acceleration cannot be explained by differences in alveolar bone architecture between *c-Fos* tg and WT mice.

**Fig. 3:**
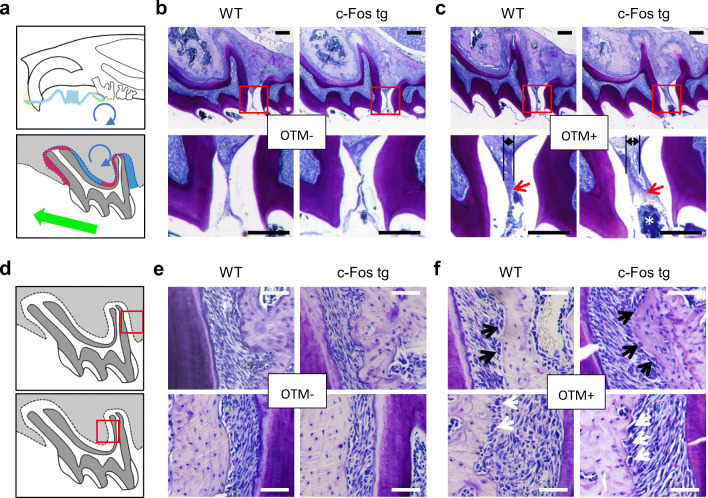
Histological analysis of OTM in *c-Fos* tg and WT mice. **a** Schematic drawing of a murine skull (upper panel) and a maxillary first molar (lower panel) illustrating the forces (green arrow) and moments (blue circle) induced by OTM. The direction of the force is mesial and intrusive, which creates different areas of compression (red) and tension (blue). **b**, **c** Toluidine-blue stained histological sections of teeth without OTM (**b**) and with OTM (**c**) of 12-week-old WT and *c-Fos* tg mice. Lower panels show magnification of the regions outlined by the red boxes. The intercoronal gaps induced by OTM (black lines) offer a retention for debris (white asterisk) resulting in a mild gingivitis (red arrows). Scale bars = 250 μm. **d** Schematic drawing of the maxillary first molar. The red boxes indicate areas of OTM-induced tension (upper panel) and pressure (lower panel) around the distal root. **e**, **f** Toluidine-blue stained histological sections of the regions indicated in **d** of teeth without OTM (**e**) and with OTM (**f**) of the same mice. Osteoblasts (black arrow) and osteoclasts (white arrows) are clearly evident after OTM. Scale bars = 50 μm

**Fig. 4:**
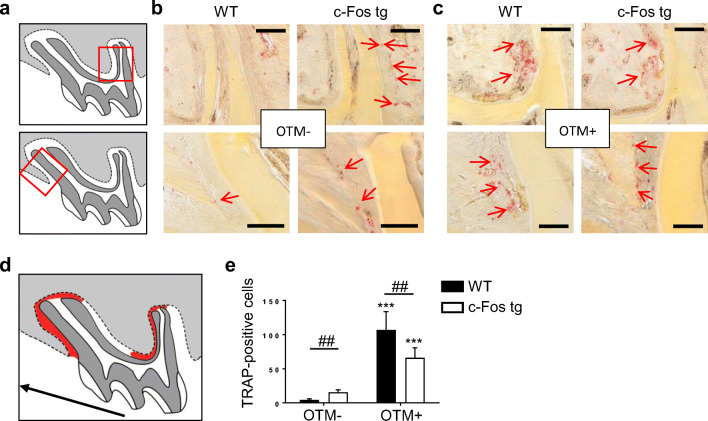
Immunohistochemical staining for osteoclasts in *c-Fos* tg and WT mice. **a** Schematic drawing of the maxillary first molar. The red boxes indicate areas of OTM-induced pressure at the distal (upper panel) and mesial (lower panel) root. **b**, **c** TRAP-stained decalcified sections of teeth without OTM (**b**) and with OTM (**c**) of 12-week-old WT and *c-Fos* tg mice. Red arrows indicate TRAP-positive cells. Scale bars = 200 μm. **d** Schematic drawing of the maxillary first molar. TRAP-positive cells were quantified in the area indicated in red. **e** Quantification of TRAP-positive cells. *n* = 4. ***P < 0.001, versus control. ##*P* < 0.01, versus wild-type

We therefore next performed a detailed histological analysis of OTM in *c-Fos* tg and WT mice (Fig. 3). In this regard, it is important to consider that OTM causes zones of compression and tension in the periodontal ligament, which have to be analysed separately (Fig. 3a). Before we looked in more detail in the histological appearance caused by OTM, we analysed toluidine-blue stained histological sections of teeth without OTM from *c-Fos* tg and WT mice (Fig. 3b). The morphology and structure of bone and teeth appeared histologically normal in *c-Fos* tg mice. We again observed that OTM caused a larger intercoronal gap in *c-Fos* tg mice as compared with controls (Fig. 3c). This was associated with food impaction causing an inflammatory epithelial thickening of the gingival papilla (Suppl. Fig. 1). We next focused on the distal root of the 1st molar, where the periodontal ligament (PDL) on the distal side is subjected to tension, whereas the PDL on the mesial side is subjected to compression (Fig. 3d). Without OTM, we found only some active bone cells in the PDL of *c-Fos* tg and control mice (Fig. 3e). The bone surface was mainly covered by non-active bone-lining cells. In contrast, after OTM numerous bone-forming osteoblasts were evident in the tensile zone of both *c-Fos* tg and control mice (Fig. 3f, upper panels). The cubic shape and arrangement of the osteoblasts clearly suggested synthesizing activity. Bone-resorbing osteoclasts were also evident on the pressure side in both *c-Fos* tg and control mice (Fig. 3f, lower panels). We further analysed the number and distribution of these osteoclasts by tartrate-resistant acid phosphatase (TRAP) staining (Fig. 4). Without OTM, we observed more TRAP-positive cells in the PDL of *c-Fos* tg as compared with WT (Fig. 4b). These TRAP-positive cells were distributed around the whole root. In contrast, OTM clearly changed the number and distribution of TRAP-positive cells both in *c-Fos* tg and WT mice as these cells were mainly found in areas of the PDL that were subjected to pressure (Fig. 4c and d). We finally quantified the number of TRAP-positive cells in the PDL of *c-Fos* tg and WT mice with and without OTM (Fig. 4e). We observed that without OTM, *c-Fos* tg mice exhibited 40% more TRAP-positive cells in the PDL as compared with WT. OTM led to a significant increase of TRAP-positive cells in both *c-Fos* tg and WT mice. However, this mechanically induced increase was less pronounced in the teeth of *c-Fos* tg mice and the number of TRAP-positive cells was therefore significantly lower after OTM in *c-Fos* tg mice as compared with that of WT mice. Taken together, our histological analysis suggests that the acceleration of tooth movement in *c-Fos* tg mice is not mediated by differences in mechanotransduction, but due to a basal increase of bone resorption.

**Fig. 5:**
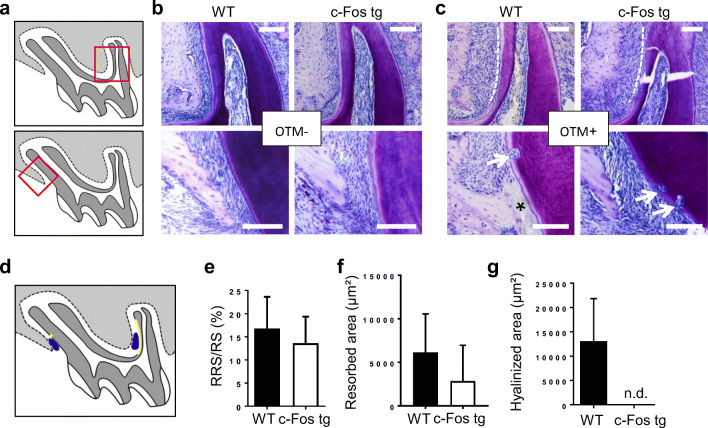
Histological analysis of OTM-induced root resorption and hyalinization in *c-Fos* tg and WT mice. **a** Schematic drawing of the maxillary first molar. The red boxes indicate areas of OTM-induced pressure at the distal (upper panel) and mesial (lower panel) root. **b**, **c** Toluidine-blue stained histological sections of teeth without OTM (**b**) and with OTM (**c**) of 12-week-old WT and *c-Fos* tg mice. Scale bars = 100 μm. Whereas lateral resorption at the distal root extends along almost the entire root surface (white dotted line), lateral resorption at the mesial root has a drop-like appearance (white arrow). Areas of hyalinization (black asterisk) were only evident in WT mice. Scale bars = 100 μm. **d** Schematic drawing of the maxillary first molar. **E**–**G** Quantification of the resorbed root surface per root surface (RRS/RS), resorbed area and hyalinized area in the PDL of 12-week-old WT and c-Fos tg mice. *n* = 4

We finally determined whether this increase of bone resorption is also associated with more root resorption in *c-Fos* tg mice. We again focused here on areas in the PDL that were subjected to orthodontic pressure (Fig. 5a). In comparison with non-stimulated teeth (OTM−), we could clearly detect root resorption in both *c-Fos* tg mice and WT mice (Fig. 5b and c). Interestingly, the shape of these resorptions differed between the two root surfaces. Whereas long, extensive resorptions were noticed at the distal root (Fig. 5c, upper panels), isolated drop-like resorption pits were noticed at the mesial root (Fig. 5c, lower panels). Quantification of root resorptions revealed no significant differences between *c-Fos* tg mice and WT mice (Fig. 5d–f). Root resorptions are usually associated with mechanically induced tissue necrosis. These cell-free, necrotic areas have a glass-like appearance on histological sections and are therefore termed areas of hyalinization [28]. Interestingly, these hyalinizations were evident to a different degree in all WT mice, but in none of the *c-Fos* tg mice (Fig. 5c and g). Taken together, our analysis suggests that the acceleration of OTM in *c-Fos* tg is not associated with more root resorption. This may be explained by the fact that OTM causes less or even no hyalinization in *c-Fos tg* mice.

## Discussion

This study shows that *c-Fos* plays an important role in the genetic control of tooth movement in vivo. We demonstrate that overexpression of *c-Fos* accelerates OTM in mice without producing more side effects such as root resorption. Since *c-Fos* overexpression did not affect alveolar bone morphology, we believe that the acceleration of OTM in *c-Fos tg* mice can be best explained by a basal increase in bone resorption and the absence of sterile necrosis (i.e. hyalinization).

In fact, the appearance of hyalinization is considered to be an important process in OTM [29]. Based on histological studies, it is assumed that hyalinization is caused by a local disturbance of blood flow in compressed PDL areas. As osteoclast differentiation is impeded in these areas, hyalinization can therefore slow down OTM. This is clearly in line with our findings in *c-Fos tg* mice, where we observed 62% faster OTM and no histological evidence of hyalinization. It remains to be established whether *c-Fos* overexpression inhibits the development of hyalinization, or whether it leads to an earlier removal of hyalinization. In this regard, it is also important to mention that *c-Fos* induces angiogenesis [18]. But regardless of this question, we believe that the absence of hyalinization is one major reason for accelerated OTM in *c-Fos tg* mice. This conclusion is further supported by experimental studies showing that surgical interventions to accelerate OTM result in less hyalinization and faster removal of hyalinized tissue [30, 31].

Another reason for faster OTM in *c-Fos* tg mice could be a basal increase in bone resorption. In fact, our histological analysis of the teeth that were not subjected to tooth movement revealed 40% more TRAP-positive cells in the PDL of *c-Fos* tg mice as compared with controls. Interestingly, the increase of osteoclastogenesis following OTM was less pronounced in *c-Fos* tg mice as compared with that in controls. One explanation for this could be that *c-Fos* transgenic cells are less sensitive to mechanical strain. Another possible explanation is that *c-Fos* can inhibit itself by negative feedback via INF-β [32]. It remains to be established whether this mechanism also takes place in PDL cells during OTM. In this regard, it is also important to mention that the load-induced expression of *c-Fos*, as an early response gene, is both rapid and short lived. Given the time frame of our experiments, it is clear that our results cannot directly be correlated to previous in vitro studies analysing the short-term response of *c-Fos* to mechanical stress [17–23]. Future studies should address this question by performing a short-term OTM in *c-Fos* tg mice following an immunohistological analysis of target genes.

One important histological observation was the occurrence of lateral root resorptions, which is a common side effect of orthodontic therapy [33]. Quantification of this root resorption in *c-Fos* tg and control mice using histomorphometry revealed no significant differences. This is an important finding as it demonstrates that the acceleration of OTM in *c-Fos* tg mice does not cause more root resorption. It was interesting to observe that the shape of lateral root resorption differed with regard to its location. Whereas root resorptions extended along almost the entire surface of the distal root, only isolated drop-like resorption pits were observed at the mesial root. A possible explanation for this could be that the tissue pressure induced by OTM differs between the mesial and the distal root. In fact, the distal surface of the distal root has a concave shape and the surrounding bone follows the root curvature. Therefore, OTM presumably creates a homogenous tissue pressure along the entire root surface. In contrast, the mesial surface of the mesial root has a convex shape and the surrounding bone does not follow entirely the root curvature, which means that the PDL is thinner at the root cervix as compared with the apical part of the root. Therefore, OTM presumably creates exceeding tissue pressure only in this cervical part. This is line with our findings as we observed only in this area root resorption and hyalinization. We believe that the correlation between root resorption and PDL morphology warrants further studies.

All these histological observations clearly demonstrate the relevance of genetically modified mice for orthodontic research. Our experimental protocol for the OTM model was based on previous studies by Taddei et al., Braga et al. and Andrade et al. [8, 9, 34–36]. According to this protocol, we bonded the Nitinol spring to the teeth using a light-curing composite. In contrast, other authors fixed the Nitinol spring with a wire ligature around the first molar [6, 7, 37, 38]. Although this might be easier to perform, we believe that wire ligatures have several disadvantages. Firstly, wire ligatures produce metal artefacts in the micro-CT scanning and these artefacts are in the interproximal area of interest. Although metal artefacts were also evident in our micro-CT scans, these were on the occlusal surface of the first molar and did not affect our quantification of the micro-CT scans. Secondly, wire ligatures can create debris niche and cause periodontal destruction during OTM. Indeed, ligatures around molars are an established model to induce periodontitis in mice [39]. Unfortunately, this ligature-induced periodontitis can also affect OTM [40]. We also noted gingival inflammation, but this inflammation was limited to the intercoronal gap and caused by the OTM and not by the appliance itself. Finally, we believe that bonding of the wire is less invasive than wire ligatures, which can cause mucous tissue injury due to the interdental threading of the wire. Accordingly, our daily weight control showed that the animals were not exposed to any serious stress. In this regard, it is also important to mention that our split-mouth designs using the contralateral side as internal controls significantly reduced the number of experimental animals as compared with other studies [41, 42].

Nevertheless, our study has certain limitations. One limitation is that our study only addresses how overexpression of *c-Fos* affects OTM. Of course, it would also be interesting to analyse whether decreased expression of *c-Fos* has an effect on OTM. However, the deletion of *c-Fos* in mice blocks osteoclastic differentiation and the dental phenotype of c-Fos-deficient mice is therefore characterized by a lack of tooth eruption and a lack of root formation [12, 43]. It is therefore not possible to perform OTM in these mice. Another limitation of our study is that *c-Fos tg* mice are characterized by the development of chondrogenic bone tumours, which may have a general effect on bone metabolism [15]. These benign tumours initially occur mainly in the tubular bones of the extremities, but with increasing age, they can also be found in the vertebral bodies and ribs [25, 27, 44–48]. It was therefore important to analyse the alveolar bone of *c-Fos tg* mice without OTM. We did not observe any of these tumours in the jaws of *c-Fos tg* mice. This finding can be explained by the fact that jaws are formed through intramembranous ossification. Furthermore, our quantification of alveolar bone loss and palatal thickness using μ-CT imaging revealed no significant differences between WT and *c-Fos tg* mice. This is an important finding as it demonstrated that differences in alveolar bone structure cannot explain the faster OTM in *c-Fos tg* mice. Finally, we would like to mention that the number of animals used for this study is comparatively low and it is possible that more significant differences would have been found with a larger sample size.

## Conclusion

Our study demonstrates that *c-Fos* overexpression in mice accelerates tooth movement due to a basal increase in bone resorption and the inhibition of mechanically induced tissue necrosis. Importantly, the faster OTM in *c-Fos tg* mice was not associated with more root resorption. We believe that this finding is also relevant with regard to surgically accelerated OTM as it demonstrates that these procedures must not result in more adverse effects such as root resorption. Future studies should use genetically modified mice to further analyse the genetic regulation of OTM.

## Electronic supplementary material

ESM 1 (PPTX 2131 kb)
