# Supplementary material for: Role of c-Fos in orthodontic tooth movement: an in vivo study using transgenic mice
Source: Clin Oral Investig. 2020 Aug 15;25(2):593–601. doi: 10.1007/s00784-020-03503-1 (PMC7819946; doi:10.1007/s00784-020-03503-1)
Supplement: Supplementary file 1 — (PPTX 2131 kb) [file 784_2020_3503_MOESM1_ESM.pptx]

## Slide 1
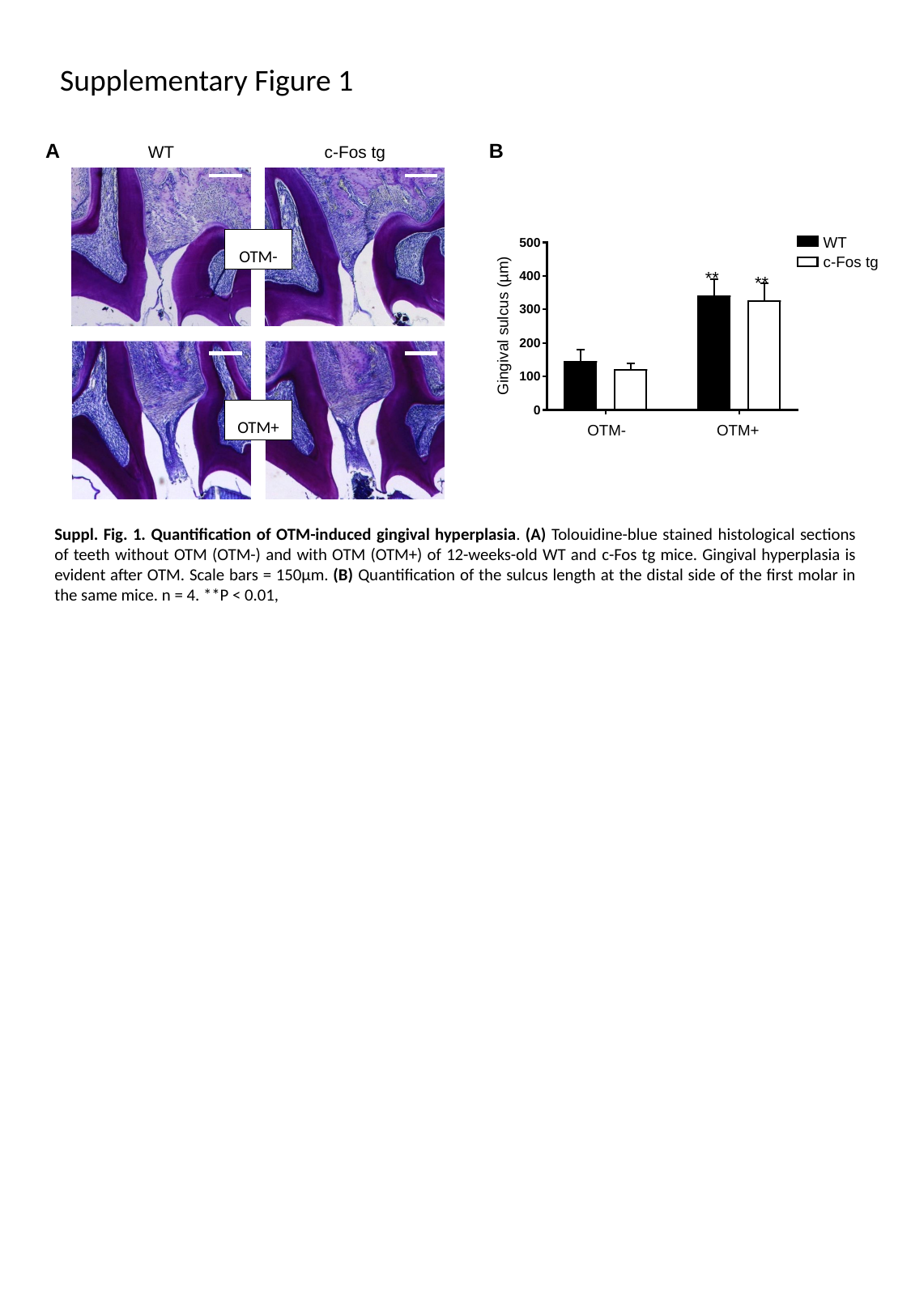

Supplementary Figure 1
A
B
c-Fos tg
WT
WT
c-Fos tg
Gingival sulcus (µm)
OTM-
OTM+
**
**
OTM-
OTM+
Suppl. Fig. 1. Quantification of OTM-induced gingival hyperplasia. (A) Tolouidine-blue stained histological sections of teeth without OTM (OTM-) and with OTM (OTM+) of 12-weeks-old WT and c-Fos tg mice. Gingival hyperplasia is evident after OTM. Scale bars = 150µm. (B) Quantification of the sulcus length at the distal side of the first molar in the same mice. n = 4. **P < 0.01,
